# Five-Year Outcomes after Paclitaxel Drug-Coated Balloon Treatment of Femoropopliteal Lesions in Diabetic and Chronic Limb-Threatening Ischemia Cohorts: IN.PACT Global Study Post Hoc Analysis

**DOI:** 10.1007/s00270-023-03478-y

**Published:** 2023-08-01

**Authors:** Michel M. P. J. Reijnen, Iris van Wijck, Marianne Brodmann, Antonio Micari, Giovanni Torsello, Seung-Woon Rha, Jeremiah Menk, Thomas Zeller, On behalf of the IN.PACT Global Study Investigators

**Affiliations:** 1 Department of Vascular Surgery, Rijnstate, Arnhem, The Netherlands; 2 Multi-Modality Medical Imaging Group, TechMed Centre, University of Twente, Enschede, The Netherlands; 3 Department of Internal Medicine, Division of Angiology, Medical University, Graz, Austria; 4 Cardiology Unit, University of Messina, Messina, Italy; 5 Institute for Vascular Research, St Franziskus-Hospital, Münster, Germany; 6 Cardiovascular Center, Korea University Guro Hospital, Seoul, Korea; 7 Medtronic, Minneapolis, MN USA; 8 Universitäts-Herzzentrum Freiburg–Bad Krozingen, Bad Krozingen, Germany

**Keywords:** Drug-coated balloon, Diabetes mellitus, Chronic limb-threatening ischemia, Clinically driven target lesion revascularization

## Abstract

**Purpose:**

To summarize the 5-year outcomes of drug-coated balloon (DCB) for the treatment of femoropopliteal lesions in patients with diabetes mellitus (DM) or chronic limb-threatening ischemia (CLTI) compared to non-DM and intermittent claudication (IC).

**Methods:**

The IN.PACT Global study was a real-world prospective, multicenter, international, single-arm study that enrolled 1535 participants. Post hoc analyses were conducted for participants with DM (*n = *560) versus non-DM (*n* = 842) and CLTI (*n* = 156) versus IC (*n* = 1246). Assessments included freedom from clinically driven target lesion revascularization (CD-TLR) through 60 months, a composite safety outcome (freedom from device- and procedure-related death through 30 days, and freedom from major target limb amputation and freedom from CD-target vessel revascularization within 60 months), and major adverse events (MAEs).

**Results:**

Kaplan–Meier estimates of 60-month freedom from CD-TLR were 67.7% and 70.5% (*p* = 0.25) in the DM and non-DM cohorts; and 60.7% and 70.5% (*p* = 0.006) in the CLTI and IC cohorts. The Kaplan–Meier 60-month composite safety outcomes were 65.1% DM versus 68.9% non-DM (*p* = 0.12); 53.2% CLTI versus 69.1% IC (*p* < 0.001). Between DM and non-DM, MAE rates were not significantly different through 60 months except for all-cause mortality which was higher in DM (23.8% versus 16.6%; *p < *0.001). Participants with CLTI had a higher cumulative incidence of major target limb amputation (6.8% versus 1.1%; *p* < 0.001) and all-cause mortality (37.4% versus 17.4%; *p* < 0.001) through 60 months compared to IC.

**Conclusions:**

In this real-world study, 5-year reintervention rates following DCB angioplasty were similar between DM and non-DM, but mortality rates were expectedly higher in patients with DM. Reintervention, mortality, and amputation rates were all higher in CLTI patients compared to IC, which is consistent with the known frailty of this patient population.

**Level of Evidence:**

Level 3, Non-randomized controlled cohort/follow-up study

**Supplementary Information:**

The online version contains supplementary material available at 10.1007/s00270-023-03478-y.

## Introduction

Revascularization plays a key role in the management of chronic limb-threatening ischemia (CLTI) patients, with or without diabetes mellitus (DM), and may also be indicated in those patients with DM suffering from lifestyle-limiting intermittent claudication (IC) and not responding to walking exercise training. The goal of revascularization is to save limbs and improve the quality of life [1, 2]. Endovascular interventions are currently recommended for all lesions less than 25 cm in length and may also be considered in patients deemed unfit for surgery [1]. Paclitaxel-coated devices have become increasingly popular for peripheral artery disease (PAD) management. In particular, paclitaxel drug-coated balloon (DCB) catheters are desirable candidates for the treatment of femoropopliteal arteries with the potential to reduce restenosis without leaving a stent behind. Multiple randomized controlled trials (RCTs) have demonstrated the safety and effectiveness of DCBs for the treatment of symptomatic femoropopliteal arterial disease compared to plain balloon angioplasty [3–11]. Single-arm prospective global studies further evaluated DCBs for real-world patients with longer, more complex lesions [12–16]. However, long-term DCB data on high-risk patient groups such as DM and CLTI are limited.

A post hoc analysis of the IN.PACT Global Study previously reported 1-year outcomes in real-world patients with CLTI treated with a paclitaxel DCB [17]. This present post hoc analysis evaluates 5-year outcomes following DCB angioplasty in IN.PACT Global Study participants with DM and CLTI compared to non-DM and IC, respectively.

## Methods

### Study Design

The real-world prospective, multicenter, international, single-arm IN.PACT Global study evaluated the safety and effectiveness of the IN.PACT Admiral DCB (Medtronic) for the treatment of atherosclerotic disease of the superficial femoral and/or popliteal artery. Sites and Principal Investigators are listed in Supplementary Table 1. Participants (*N* = 1535) were enrolled across 64 international sites from 2012 to 2014, of which 1406 participants were treated with the IN.PACT Admiral DCB and included in the clinical cohort that was used for the current analysis. Detailed study design and outcomes through 5 years have been reported previously [12–14, 18].

This post hoc analysis reports two cohorts 1) DM versus non-DM and 2) CLTI (Rutherford category [RC] 4 and 5) versus IC (RC 2 and 3). Of note, enrollment of patients with RC 5 (*n* =  36) was considered a protocol deviation in the study. Additionally, one RC1 participant was enrolled as a protocol deviation.

Participants were followed at discharge, 30 days, 6 months, 12 months and then annually through 60 months. Follow-up evaluations were conducted via clinical visits through 36 months and by phone at 48 and 60 months. To verify mortality information, investigational sites were asked to obtain vital status updates from participants who withdrew or were lost to follow-up. Vital status update results are labeled as such when included.

An independent Clinical Events Committee (CEC; Syntactx, New York, NY, USA) adjudicated all major adverse events (MAEs) including clinically driven target lesion revascularizations (CD-TLRs) and clinically driven target vessel revascularizations (CD-TVRs) through 60 months after the index procedure. The study was conducted in accordance with good clinical practice guidelines, the Declaration of Helsinki and all applicable country laws. The institutional review board or ethics committee at each participating site approved the study protocol. Informed consent was obtained from all participants prior to enrollment. The trial was registered on the National Institutes of Health website (*ClinicalTrials.gov* identifier: NCT01609296).

### Outcome Measures

Freedom from CD-TLR was reported through 60 months. CD-TLR and CD-TVR were defined as any reintervention within the target lesion(s) or vessel(s), respectively, because of symptoms or drop of ankle-brachial index (ABI) of ≥20% or >0.15 when compared with post-index procedure baseline ABI. The composite safety outcome was defined as freedom from device- and procedure-related death through 30 days and freedom from major target limb amputation and CD-TVR within 60 months after the index procedure. Other assessments through 60 months included any TLR, any TVR, and the incidence of MAEs (all-cause mortality, CD-TVR, major target limb amputation, and target lesion thrombosis). Functional outcomes including primary and secondary sustained clinical improvement were reported through 36 months. Full definitions of outcome measures are described in the Supplementary Methods.

### Statistics

All analyses were based on participants with evaluable data. Baseline demographics, clinical characteristics, and outcomes are reported or analyzed on a participant basis. Lesion and device characteristics are reported on a lesion and device basis, respectively. Data are summarized descriptively using percentages and frequencies for categorical variables and the mean, standard deviation (SD), and number of observations for continuous variables. Time-to-event outcomes are summarized with survival curves and survival probabilities using the Kaplan–Meier method with log-rank *P* values. Confidence intervals (95% CI) were derived for time-to-event outcomes using the log-log transformation. Outcomes are also described using the restricted mean survival time (RMST) with a time horizon of 1800 days and 95% CI without bias correction. A participant was considered part of the analysis set if the study DCB was introduced into the sheath, after the guidewire had successfully passed through the target lesion. Annual cutoffs used 360 days per year (e.g., 1800 days for the 5-year cut-off). Statistical significance was set at 0.05. Statistical analyses were performed using SAS version 9.4 (SAS Institute, Cary, NC).

## Results

### Patient Population

#### DM Versus Non-DM

**Fig. 1:**
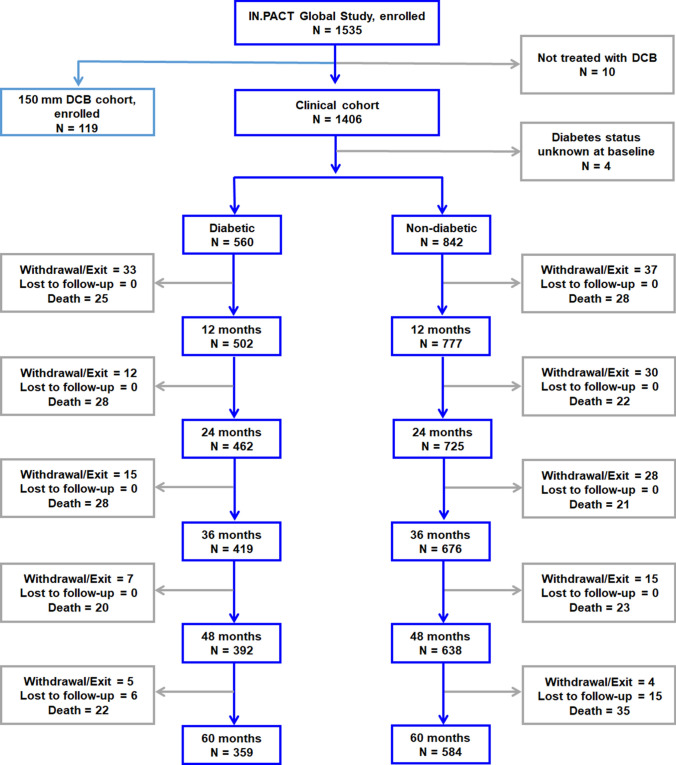
Participant flowchart of the diabetic and non-diabetic cohorts in the IN.PACT Global Study. Four participants in the clinical cohort did not have known DM status at baseline.

A participant flowchart is shown in Fig. 1. A total of 1402 participants with known DM status were stratified into the DM (*n = *560) and non-DM (*n = *842) cohorts. Overall, 60-month follow-up compliance was 96.4% and 97.4% for DM and non-DM, respectively. Participants in the DM cohort had higher burdens of obesity, hypertension, hyperlipidemia, coronary and carotid artery disease, renal insufficiency, concomitant below-the-knee disease, advanced PAD, and previous limb amputation (major or minor) as compared to the non-DM cohort (Table 1). The baseline lesion and procedural characteristics were similar between groups (Table 2), except for a higher calcification burden, including more severe calcification, in the DM (12.4%) compared to the non-DM cohort (8.7%). The mean lesion length was equivalent between DM and non-DM. Provisional stenting rates were 18.7% DM and 23.0% non-DM (*p *= 0.03).

**Table 1:** Baseline demographic and clinical characteristics in participants with diabetes mellitus and non-diabetes mellitus*

Participant characteristics	Diabetes mellitus (*N* = 560 participants) (*N*= 603 limbs)	Non-diabetes mellitus (*N* = 842 participants) (*N* = 914 limbs)	*p*-value
Age (years)	68.5 ± 9.4 (554)	68.6 ± 10.5 (838)	0.81
BMI ≥ 30 kg/m^2^	27.8 ± 4.8 (550)	26.0 ± 4.1 (837)	< 0.001
Obesity (BMI ≥ 30 kg/m^2^)	27.5% (151/550)	16.0% (134/837)	< 0.001
Male	67.7% (379/560)	67.9% (572/842)	0.95
Hypertension	89.4% (500/559)	79.4% (666/839)	< 0.001
Hyperlipidemia	74.9% (409/546)	67.4% (548/813)	0.003
Diabetes mellitus	100.0% (560/560)	0.0% (0/842)	< 0.001
Insulin-dependent diabetes mellitus	44.5% (249/560)	0.0% (0/842)	< 0.001
Carotid artery disease	24.6% (115/468)	17.2% (125/725)	0.002
Coronary heart disease	50.6% (265/524)	34.0% (274/805)	< 0.001
Current smoker	23.0% (129/560)	37.6% (317/842)	< 0.001
Renal insufficiency (baseline serum creatinine ≥ 1.5 mg/dl)	16.8% (84/500)	7.3% (52/712)	< 0.001
On dialysis	5.2% (29/555)	1.1% (9/837)	< 0.001
Below-the-knee vascular disease of target leg (stenotic/occluded)	55.9% (289/517)	38.6% (305/791)	< 0.001
Previous peripheral revascularization	54.5% (305/560)	51.1% (430/842)	0.23
Iliac	15.2% (85/560)	18.4% (155/842)	0.13
Common femoral	6.6% (37/560)	6.4% (54/842)	0.91
Femoral profunda	2.1% (12/560)	2.1% (18/842)	> 0.99
Superficial femoral	44.8% (251/560)	41.1% (346/842)	0.17
Popliteal	20.4% (114/560)	12.0% (101/842)	< 0.001
Below-the-knee	10.9% (61/560)	7.1% (60/842)	0.02
Other location	0.9% (5/560)	1.1% (9/842)	> 0.99
Previous limb amputation	8.0% (45/560)	1.0% (8/842)	< 0.001
Toe	5.5% (31/560)	0.4% (3/842)	< 0.001
Transmetatarsal	1.3% (7/560)	0.1% (1/842)	0.008
Below-the-knee	1.1% (6/560)	0.2% (2/842)	0.07
Above-the-knee	0.5% (3/560)	0.2% (2/842)	0.39
Rutherford category			0.003
1	0.0% (0/559)	0.1% (1/840)^†^	
2	29.0% (162/559)	32.6% (274/840)	
3	55.8% (312/559)	58.8% (494/840)	
4	11.1% (62/559)	6.9% (58/840)	
5	4.1% (23/559)^†^	1.5% (13/840)^†^	
ABI (mmHg ratio), per target limb	0.7 ± 0.2 (543)	0.7 ± 0.2 (845)	0.06

Site-reported data

Continuous data are presented as the mean ± standard deviation (number of participants or limbs with data); categorical data are given as the percentage (number/number of participants with data). All data are participant based unless otherwise stated

*Summaries are based on non-missing assessments. In some cases, baseline demographic or clinical data were not available

^†^Participants with Rutherford Category 1 and 5 were enrolled and included in this analysis due to protocol violation

*ABI*, ankle-brachial index; *BMI*, body-mass index

**Table 2:** Lesion and procedural characteristics in participants with diabetes mellitus and non-diabetes mellitus*

	Diabetes mellitus (*N* = 560 participants) (*N* = 722 lesions)	Non-diabetes mellitus (*N* = 842 participants) (*N* = 1048 lesions)	*p*-value
*Baseline lesion characteristics*			
Lesion location^†^			
SFA proximal	30.7% (222/722)	33.2% (348/1048)	0.30
SFA middle	52.1% (376/722)	50.6% (530/1048)	0.56
SFA distal	52.6% (380/722)	57.5% (603/1048)	0.046
PA	28.7% (207/722)	26.2% (275/1048)	0.28
P1	21.6% (156/722)	20.0% (210/1048)	0.44
P2	14.1% (102/722)	12.3% (129/1048)	0.28
P3	5.4% (39/722)	4.3% (45/1048)	0.31
Lesion type			
De novo	72.9% (526/722)	75.2% (788/1048)	0.27
Restenotic (non-stented)	8.7% (63/722)	7.0% (73/1048)	0.17
In-stent restenosis	18.4% (133/722)	17.8% (187/1048)	0.75
Vessel^†^			
SFA	87.0% (628/722)	88.1% (923/1048)	0.51
PA	28.7% (207/722)	26.2% (275/1048)	0.28
Calcification			< 0.001
None	28.2% (203/720)	33.5% (351/1048)	
Mild	25.3% (182/720)	30.1% (315/1048)	
Moderate	20.4% (147/720)	17.2% (180/1048)	
Moderately severe	13.8% (99/720)	10.6% (111/1048)	
Severe	12.4% (89/720)	8.7% (91/1048)	
Thrombus	0.4% (3/722)	1.0% (11/1048)	0.18
RVD (mm)	5.2 ± 0.7 (722)	5.2 ± 0.7 (1048)	0.10
Chronic total occlusion	31.3% (226/722)	38.3% (401/1048)	0.003
Diameter stenosis (%)	87.9 ± 11.9 (722)	89.4 ± 12.5 (1048)	0.01
Lesion length (cm)	12.1 ± 9.3 (722)	12.1 ± 9.7 (1048)	0.88
*Procedural characteristics*			
Number of bilateral participants	7.7% (43/560)	8.6% (72/842)	0.62
Nights in hospital for index procedure	2.5 ± 7.6 (560)	1.7 ± 2.3 (842)	0.02
Pre-dilatation	75.4% (422/560)	79.7% (671/842)	0.06
Post-dilatation	32.5% (180/553)	36.8% (309/840)	0.11
Provisional stent rate per lesion	18.7% (133/713)	23.0% (240/1045)	0.03
Spot stenting	24.8% (33/133)	24.2% (58/240)	0.90
Partial lesion coverage	36.8% (49/133)	38.3% (92/240)	0.82
Whole lesion coverage	38.3% (51/133)	37.5% (90/240)	0.91
Reason for provisional stenting			
Persistent residual stenosis ≥ 50%	57.1% (76/133)	60.4% (145/240)	0.58
>10 mmHg trans lesion gradient	0.8% (1/133)	0.4% (1/240)	>0.99
Flow-limiting dissection	54.1% (72/133)	53.3% (128/240)	0.91
Other	3.8% (5/133)	5.8% (14/240)	0.47
*Post-procedure characteristics*			
Geographic miss	1.7% (12/722)	1.5% (16/1048)	0.85
Dissection grade			
0 (no dissection)	60.3% (435/721)	54.4% (570/1048)	0.02
A	14.3% (103/721)	14.4% (151/1048)	> 0.99
B	12.5% (90/721)	14.9% (156/1048)	0.16
C	5.8% (42/721)	7.9% (83/1048)	0.11
D	4.4% (32/721)	4.4% (46/1048)	> 0.99
E	2.2% (16/721)	3.2% (34/1048)	0.24
F	0.4% (3/721)	0.8% (8/1048)	0.54
Residual stenosis (%)	11.8 ± 12.1 (713)	11.3 ± 11.6 (1044)	0.38
Total target lesion length treated with study device (cm)	14.6 ± 9.4 (713)	14.64 ± 9.78 (1045)	0.98
*Acute outcomes*			
Immediate hemodynamic improvement at post-index procedure	88.1% (424/481)	88.9% (658/740)	0.71
Device success	99.2% (1200/1210)	99.6% (1781/1789)	0.23
Procedural success	99.3% (708/713)	99.4% (1038/1044)	0.77
Clinical success	98.2% (543/553)	98.8% (829/839)	0.36

Site reported data

Continuous data are presented as the mean ± standard deviation (observations with data); categorical data are given as the percentage (number/observations with data). Definitions are described in Methods and Supplementary Methods

*Summaries are based on non-missing assessments. In some cases, baseline demographic or clinical data were not available

^†^Multiple lesion locations are reported in a single target limb, the total lesion locations could be more than the total number of target limbs

*PA*, popliteal artery; *RVD*, reference vessel diameter; *SFA*, superficial femoral artery

#### IC Versus CLTI

**Fig. 2:**
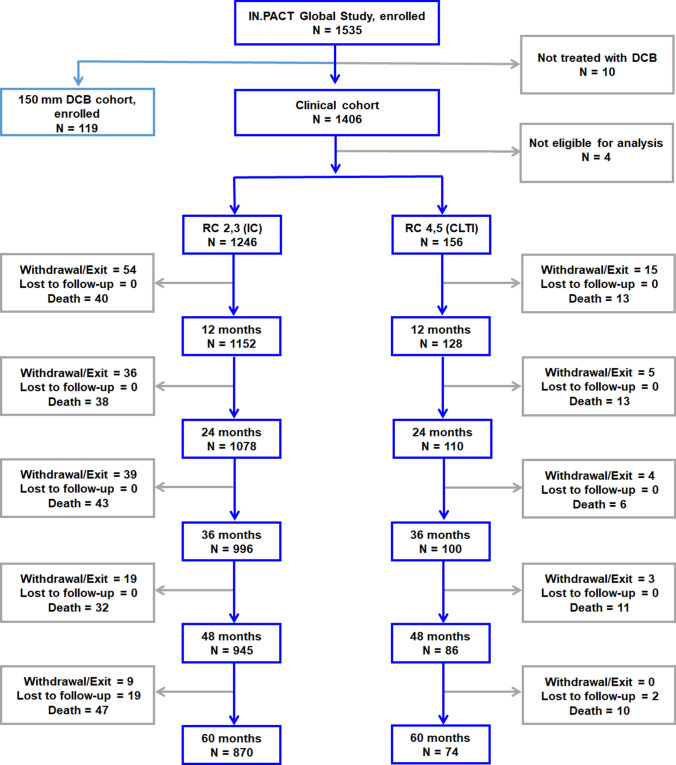
Participant flowchart of IC and CLTI cohorts at baseline in the IN.PACT Global Study. Four participants in the clinical cohort were not eligible for this analysis: RC was not known for three participants and one participant was in RC 1 at baseline. CLTI, chronic limb-threatening ischemia; IC, intermittent claudication, RC, Rutherford category.

The flowchart for participants with CLTI (RC 4,5) and IC (RC 2,3) is shown in Fig. 2. Of the 1406 participants, 3 did not have known RC and 1 participant was in RC 1 at baseline. The remaining 1402 with known baseline RC and treated with the DCB were stratified into the CLTI (*n = *156) and IC (*n* = 1246) cohorts. Overall follow-up compliance at 60 months was 94.6% in the CLTI cohort and 97.4% in the IC cohort. Participants in the CLTI cohort were significantly older, were more often women, had higher burdens of DM, renal insufficiency, concomitant below-the-knee vascular disease, previous limb amputation, and had lower ABI compared to the IC cohort (Table 3). There were also significant differences in the lesion characteristics (Table 4): compared to IC, CLTI participants had more popliteal involvement, higher calcification burden, smaller reference vessel diameter, and longer lesions (13.9±10.6 cm versus 11.9±9.4 cm; *p = *0.01). Provisional stenting rates were similar between CLTI and IC.

**Table 3:** Baseline demographic and clinical characteristics in participants with IC and CLTI*

Participant characteristics	CLTI (*N* = 156 participants) (*N* = 163 limbs)	IC (*N* = 1246 participants) (*N* = 1354 limbs)	*p*-value
Age (years)	71.8 ± 10.4 (155)	68.2 ± 10.0 (1237)	< 0.001
BMI ≥ 30 kg/m^2^	26.1 ± 5.1 (152)	26.8 ± 4.4 (1236)	0.11
Obesity (BMI ≥ 30 kg/m^2^)	18.4% (28/152)	20.7% (256/1236)	0.59
Male	55.8% (87/156)	69.3% (864/1246)	0.001
Hypertension	85.3% (133/156)	83.2% (1032/1241)	0.57
Hyperlipidemia	62.3% (96/154)	71.5% (862/1205)	0.02
Diabetes mellitus	54.5% (85/156)	38.2% (474/1242)	< 0.001
Insulin-dependent diabetes mellitus	28.8% (45/156)	16.3% (203/1242)	< 0.001
Carotid artery disease	17.8% (21/118)	20.4% (220/1076)	0.55
Coronary heart disease	44.0% (62/141)	40.1% (476/1188)	0.41
Current smoker	22.4% (35/156)	33.0% (411/1246)	0.008
Renal insufficiency (baseline serum creatinine ≥ 1.5 mg/dl)	20.1% (28/139)	10.1% (108/1074)	< 0.001
On dialysis	10.3% (16/156)	1.8% (22/1236)	< 0.001
Below-the-knee vascular disease of target leg (stenotic/occluded)	63.3% (93/147)	43.1% (499/1159)	< 0.001
Previous peripheral revascularization	55.8% (87/156)	51.9% (647/1246)	0.40
Iliac	13.5% (21/156)	17.6% (219/1246)	0.22
Common femoral	9.0% (14/156)	6.2% (77/1246)	0.17
Femoral profunda	2.6% (4/156)	2.0% (25/1246)	0.56
Superficial femoral	42.9% (67/156)	42.5% (529/1246)	0.93
Popliteal	24.4% (38/156)	14.2% (177/1246)	0.002
Below-the-knee	19.2% (30/156)	7.3% (91/1246)	< 0.001
Other location	2.6% (4/156)	0.8% (10/1246)	0.06
Previous limb amputation	16.0% (25/156)	2.2% (28/1246)	< 0.001
Toe	9.6% (15/156)	1.5% (19/1246)	< 0.001
Transmetatarsal	1.9% (3/156)	0.4% (5/1246)	0.05
Below-the-knee	3.8% (6/156)	0.2% (2/1246)	< 0.001
Above-the-knee	1.3% (2/156)	0.2% (3/1246)	0.10
Rutherford category			< 0.001
1	0.0% (0/156)	0.0% (0/1246)	
2	0.0% (0/156)	35.0% (436/1246)	
3	0.0% (0/156)	65.0% (810/1246)	
4	76.9% (120/156)	0.0% (0/1246)	
5	23.1% (36/156)^†^	0.0% (0/1246)	
ABI (mmHg ratio), per target limb	0.6 ± 0.3 (144)	0.7 ± 0.2 (1245)	< 0.001

Site reported data

Continuous data are presented as the mean ± standard deviation (number of participants or limbs with data); categorical data are given as the percentage (number/number of participants or limbs with data). All data are participant based otherwise stated

*Summaries are based on non-missing assessments. In some cases, baseline demographic or clinical data were not available

^†^Participants with Rutherford Category 5 were enrolled and included in this analysis due to protocol violation

ABI, ankle-brachial index; BMI, body-mass index; CLTI, chronic limb-threatening ischemia; IC, intermittent claudication

**Table 4:** Lesion and procedural characteristics in participants with IC and CLTI*

	CLTI (*N* = 156 participants) (*N* = 194 lesions)	IC (*N* = 1246 participants) (*N* = 1574 lesions)	*p*-value
*Baseline lesion characteristics*			
Lesion location^†^			
SFA proximal	32.5% (63/194)	32.1% (505/1574)	0.94
SFA middle	46.9% (91/194)	51.7% (814/1574)	0.22
SFA distal	63.9% (124/194)	54.5% (858/1574)	0.01
PA	41.8% (81/194)	25.5% (402/1574)	< 0.001
P1	32.5% (63/194)	19.3% (304/1574)	< 0.001
P2	22.7% (44/194)	11.9% (187/1574)	< 0.001
P3	9.8% (19/194)	4.1% (65/1574)	0.002
Vessel^†^			
SFA	86.1% (167/194)	87.7% (1381/1574)	0.49
PA	41.8% (81/194)	25.5% (402/1574)	< 0.001
Lesion type			0.97
De novo	74.2% (144/194)	74.4% (1171/1574)	
Restenotic (non-stented)	8.8% (17/194)	7.5% (118/1574)	
In-stent restenosis	17.0% (33/194)	18.1% (285/1574)	
Calcification			0.03
None	23.2% (45/194)	32.3% (508/1572)	
Mild	32.0% (62/194)	27.7% (436/1572)	
Moderate	19.1% (37/194)	18.4% (290/1572)	
Moderately severe	14.4% (28/194)	11.4% (179/1572)	
Severe	11.3% (22/194)	10.1% (159/1572)	
Thrombus	2.6% (5/194)	0.6% (9/1574)	0.01
RVD (mm)	5.0 ± 0.7 (194)	5.2 ± 0.7 (1574)	< 0.001
Chronic total occlusion	41.2% (80/194)	34.8% (548/1574)	0.08
Diameter stenosis (%)	89.4 ± 12.2 (194)	88.8 ± 12.3 (1574)	0.53
Lesion length (cm)	13.9 ± 10.6 (194)	11.9 ± 9.4 (1574)	0.01
*Procedural characteristics*			
Number of bilateral participants	4.5% (7/156)	8.7% (108/1246)	0.09
Nights in hospital for index procedure	3.7 ± 8.7 (156)	1.8 ± 4.5 (1246)	0.01
Pre-dilatation	75.0% (117/156)	78.5% (978/1246)	0.36
Post-dilatation	34.4% (53/154)	35.2% (436/1239)	0.93
Provisional stent rate per lesion	20.3% (39/192)	21.4% (334/1564)	0.78
Spot stenting	33.3% (13/39)	23.4% (78/334)	0.17
Partial lesion coverage	23.1% (9/39)	39.5% (132/334)	0.05
Whole lesion coverage	43.6% (17/39)	37.1% (124/334)	0.49
Reason for provisional stenting			
Persistent residual stenosis ≥ 50%	66.7% (26/39)	58.4% (195/334)	0.39
>10 mmHg trans lesion gradient	2.6% (1/39)	0.3% (1/334)	0.20
Flow-limiting dissection	59.0% (23/39)	53.0% (177/334)	0.50
Other	2.6% (1/39)	5.4% (18/334)	0.71
*Post-procedure characteristics*			
Geographic miss	3.6% (7/194)	1.4% (22/1574)	0.03
Dissection grade			
0 (no dissection)	65.5% (127/194)	55.6% (875/1573)	0.009
A	15.5% (30/194)	14.2% (223/1573)	0.66
B	8.8% (17/194)	14.7% (231/1573)	0.03
C	5.2% (10/194)	7.3% (115/1573)	0.30
D	2.6% (5/194)	4.6% (73/1573)	0.26
E	1.0% (2/194)	3.1% (48/1573)	0.16
F	1.5% (3/194)	0.5% (8/1573)	0.11
Residual stenosis (%)	10.1 ± 11.0 (192)	11.6 ± 11.9 (1563)	0.08
Total target lesion length treated with study device (cm)	16.1 ± 10.6 (192)	14.5 ± 9.5 (1564)	0.04
*Acute outcomes*			
Immediate hemodynamic improvement at post-index procedure	90.2% (119/132)	88.5% (965/1091)	0.66
Device success	99.7% (352/353)	99.4% (2625/2642)	0.71
Procedural success	100.0% (192/192)	99.3% (1552/1563)	0.62
Clinical success	98.1% (151/154)	98.6% (1221/1238)	0.48

Site reported data

Continuous data are presented as the mean ± standard deviation (observations with data); categorical data are given as the percentage (number/observations with data). Definitions are described in Methods and Supplementary Methods

*Summaries are based on non-missing assessments. In some cases, baseline demographic or clinical data were not available

^†^Multiple lesion locations are reported in a single target limb, the total lesion locations could be more than the total number of target limbs

*CLTI*, chronic limb-threatening ischemia; *PA*, popliteal artery; *IC*, intermittent claudication; *RVD*, reference vessel diameter

### Follow-up Outcomes

#### DM Versus Non-DM

**Fig. 3:**
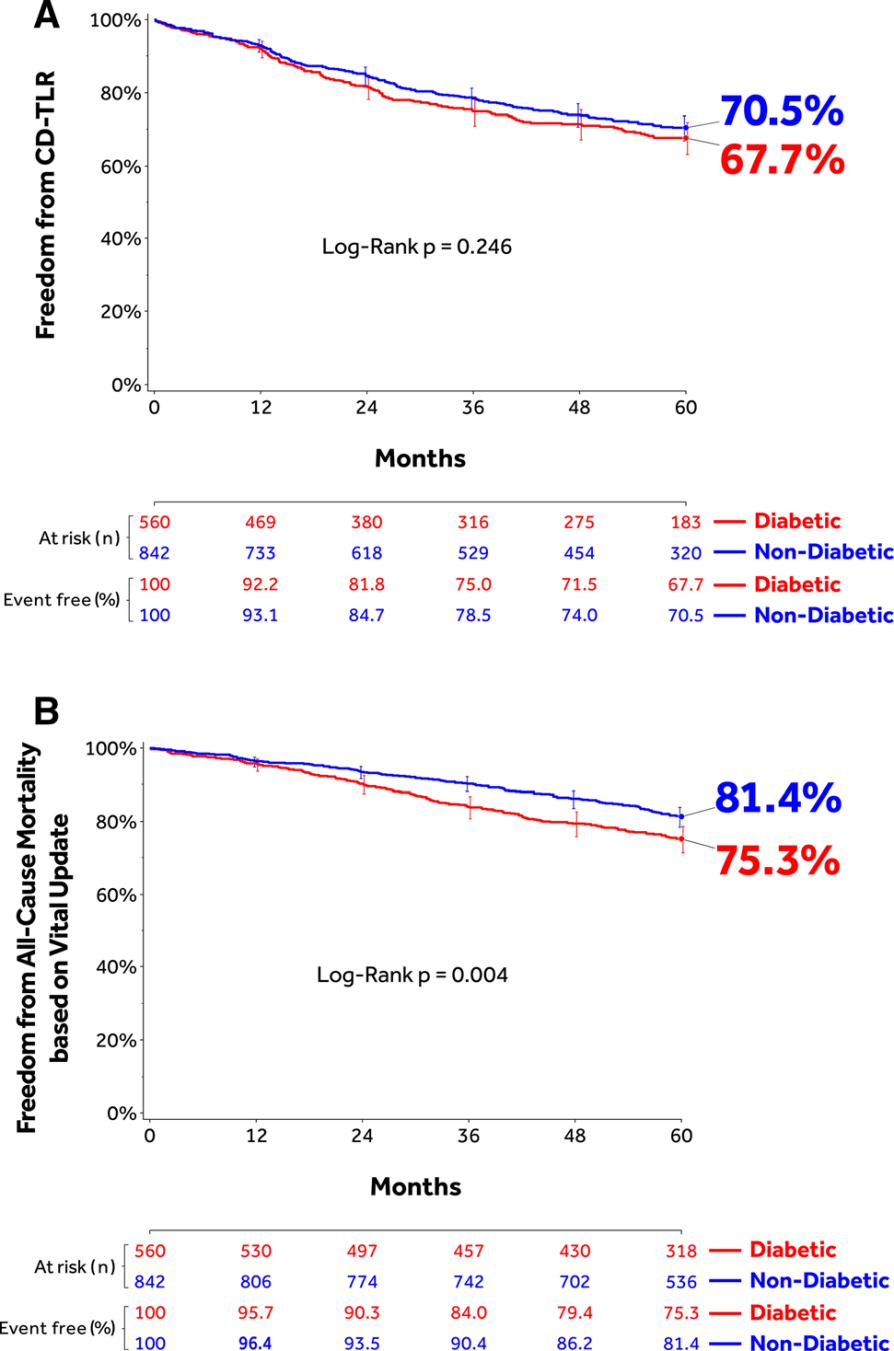
**(A)** Kaplan–Meier estimate of freedom from clinically driven target lesion revascularization (CD-TLR) through 1800 days (60 months), and **(B)** Kaplan–Meier estimate of freedom from all-cause mortality through 1800 days (60 months) in the IN.PACT Global Study diabetic and non-diabetic cohorts treated with the IN.PACT Admiral DCB. Bars represent the 95% confidence intervals.

Freedom from CD-TLR through 60 months was 67.7% (95% CI: 63.2–71.8%) in DM participants compared to 70.5% (95% CI: 66.9–73.7%) in non-DM participants (*p = *0.25) (Fig. 3A). The RMST to first CD-TLR was not significantly different between cohorts (Table 5). Primary and secondary sustained clinical improvement rates were available through 36 months and were significantly lower in the DM cohort compared to the non-DM cohort (Table 5). The 60-month Kaplan–Meier composite safety outcomes were not significantly different between DM and non-DM participants: 65.1% (95% CI: 60.5–69.3%) DM versus 68.9% (95% CI: 65.3–72.2%) non-DM; *p *= 0.12 (Table 5). Compared to the non-DM cohort, the DM cohort had a higher cumulative incidence of composite major adverse events through 60 months (49.8% [95% CI: 45.5–54.3%] versus 43.3% [95% CI: 39.8–46.9%]; *p * = 0.009) driven by a higher all-cause death rate (23.8% versus 16.6%; p < 0.001). The rates of the individual MAE components are shown in Table 5. The survival probability of all-cause mortality based on vital status update (after accounting for participants who withdrew or were lost to follow-up) was 75.3% (95% CI: 71.4–78.7%) in the DM cohort and 81.4% (95% CI: 78.5–83.9%) in the non-DM cohort (*p * = 0.004) (Fig. 3B).

**Table 5:** Outcomes through 60 months by diabetes status

Parameters	Diabetes mellitus (N = 560 participants)	Non-diabetes mellitus (N = 842 participants)	*p*-value
*Safety parameters*			
Composite safety outcome – freedom from:	65.1%	68.9%	0.12
Device- and procedure-related death through 30 days	0.4% (2)	0.1% (1)	0.35
Major target limb amputation within 60 months	2.5% (11)	1.1% (8)	0.09
CD-TVR within 60 months	33.6% (157)	30.8% (223)	0.24
*Cumulative complications within 60 months*			
MAE composite	49.8% (256)	43.3% (331)	0.009
Death (all-cause)	23.8% (119)	16.6% (124)	< 0.001
CD-TVR	33.6% (157)	30.8% (223)	0.24
Major target limb amputation	2.5% (11)	1.1% (8)	0.09
Thrombosis	4.9% (25)	6.1% (47)	0.41
CD-TLR	32.3% (151)	29.5% (214)	0.25
Any TVR	35.0% (163)	31.2% (226)	0.14
Any TLR	33.6% (157)	29.8% (216)	0.13
*Other major secondary endpoints*			
Restricted survival time to first CD-TLR (days) through 60 months	1445.0 ± 26.0* (151)	1486.6 ± 19.8* (214)	0.20
Primary sustained clinical improvement at 36 months	54.2% (215/397)	63.9% (389/609)	0.002
Secondary sustained clinical improvement at 36 months	74.5% (274/368)	85.2% (485/569)	< 0.001
Sustained hemodynamic improvement at 36 months	40.3% (139/345)	53.7% (297/553)	< 0.001
Change in health status by EQ-5D Index to 36 months	0.130 ± 0.360 (308)	0.132 ± 0.315 (513)	0.93
Walking impairment by WIQ to 36 months	71.3 ± 31.1 (309)	76.4 ± 30.1 (520)	0.02
Nights in hospital due to index lesion to 36 months	3.7 ± 11.2 (506)	2.5 ± 4.1 (842)	0.02

For clinical safety endpoints, percentages are cumulative incidence based on the Kaplan–Meier Estimate (number of patients with events). Categorical data are given as the percentage (number/observations with data). Continuous data are presented as the mean ± standard deviation with the sample size unless otherwise stated. Adverse events were adjudicated by the independent Clinical Events Committee, all duplex ultrasound and angiographic measures were made by the independent core laboratories, and all other data were site reported. Definitions are described in Methods and Supplementary Methods

*WIQ*, Walking impairment questionnaire

*Mean ± standard error

#### DM Subset Analysis

There was no significant difference in the 60-month cumulative incidence of CD-TLR (35.1% versus 30.4%; *p* = 0.53) or major target limb amputation (3.8% versus 1.6%; *p* = 0.16) between the insulin-dependent DM and non-insulin-dependent DM sub-cohorts. The cumulative incidence of all-cause mortality with vital status was higher in the insulin-dependent DM sub-cohort compared to non-insulin-dependent DM sub-cohort (30.9% versus 19.9%; *p* = 0.003) (Supplementary Fig. 1).

#### CLTI Versus IC

**Fig. 4:**
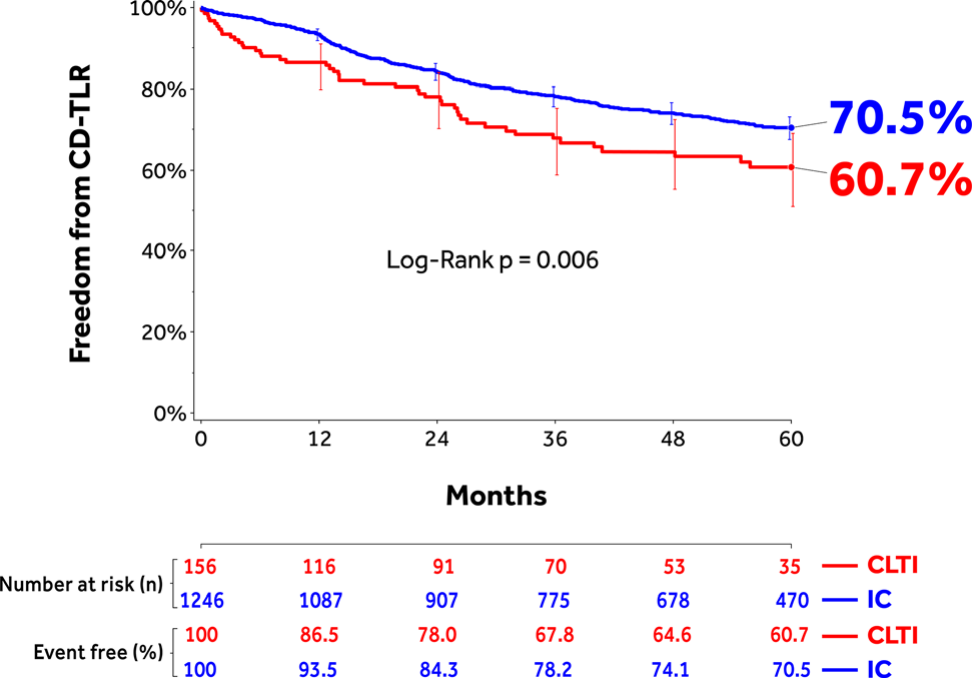
Kaplan–Meier estimate of freedom from CD-TLR through 1800 days (60 months) in the IN.PACT Global Study IC and CLTI Cohorts treated with the IN.PACT Admiral DCB. Bars represent the 95% confidence intervals. CD-TLR, clinically driven target lesion revascularization; CLTI, chronic limb-threatening ischemia; IC, intermittent claudication.

**Fig. 5:**
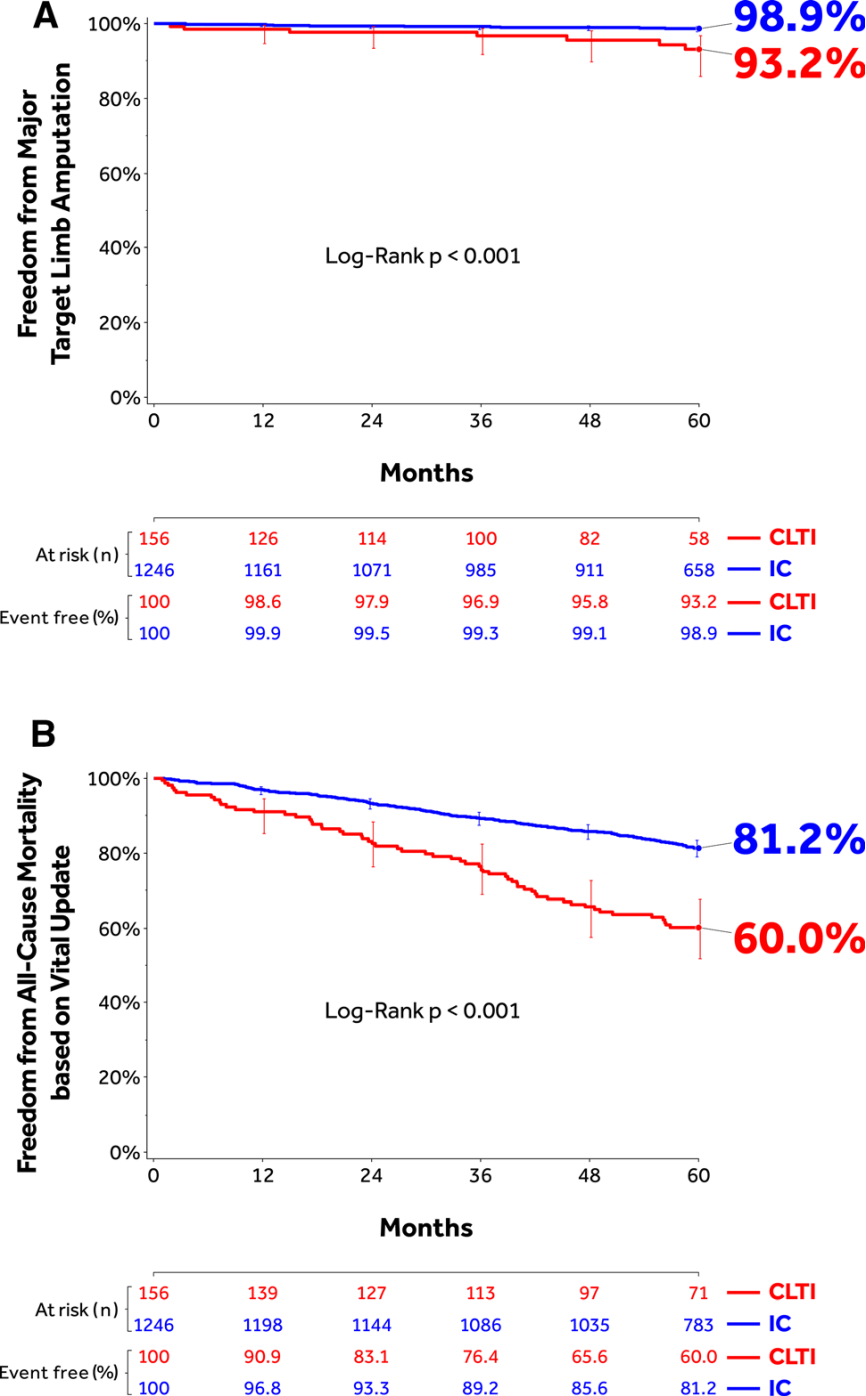
**(A)** Kaplan–Meier estimate of freedom from major target limb amputation 1800 days (60 months) and **(B)** Kaplan–Meier estimate of freedom from all-cause mortality after vital status update through 1800 days (60 months) in the IN.PACT Global Study IC and CLTI cohorts treated with the IN.PACT Admiral DCB. Bars represent the 95% confidence intervals. CLTI, chronic limb-threatening ischemia; IC, intermittent claudication.

Freedom from CD-TLR through 60 months was significantly lower in the CLTI cohort (60.7%; 95% CI: 50.9–69.1%) compared to the IC cohort (70.5%; 95% CI: 67.6–73.2%; *p* = 0.006) (Fig. 4). The RMST to first CD-TLR was lower in CLTI versus IC (Table 6). Primary sustained clinical improvement through 36 months was lower in the CLTI cohort. However, no statistically significant difference was observed for secondary sustained clinical improvement between the two cohorts (Table 6). The composite safety outcome was significantly better in the IC cohort compared to CLTI (53.2% [95% CI: 43.5–62.0%] CLTI versus 69.1% [95% CI: 66.2–71.8%] IC; *p* < 0.001) (Table 6). The cumulative incidence of 60-month composite MAE was 65.4% (95% CI: 57.3–73.3%) CLTI versus 43.5% (94% CI: 40.6–46.4%) IC (*p* < 0.001) (Table 6). Rates of individual MAE components are shown in Table 6. Freedom from major target limb amputation was 93.2% (95% CI: 85.9–96.8%) and 98.9% (95% CI: 98.0–99.4%) in the CLTI and IC cohorts, respectively (*p* < 0.001) (Fig. 5A). The freedom from all-cause mortality with vital status update was 60.0% (95% CI: 51.7–67.4%) and 81.2% (95% CI: 78.9–83.3%) in the CLTI and IC cohorts, respectively (*p* < 0.001) (Fig. 5B).

**Table 6:** Outcomes through 60 months in IC and CLTI participants

Parameters	CLTI (*N* = 156 participants)	IC (*N* = 1246 participants)	*p*-value
*Safety parameters*			
Composite safety outcomes–freedom from:	53.2%	69.1%	< 0.001
Device- and procedure-related death through 30 days	0.6% (1)	0.2% (2)	0.22
Major target limb amputation within 60 months	6.8% (7)	1.1% (12)	< 0.001
CD-TVR within 60 months	43.1% (52)	30.5% (327)	0.001
*Cumulative complications within 60 months*			
MAE composite	65.4% (92)	43.5% (495)	< 0.001
Death (all-cause)	37.4% (50)	17.4% (194)	< 0.001
CD-TVR	43.1% (52)	30.5% (327)	0.001
Major target limb amputation	6.8% (7)	1.1% (12)	< 0.001
Thrombosis	9.2% (12)	5.3% (60)	0.07
CD-TLR	39.3% (48)	29.5% (316)	0.006
Any TVR	43.1% (52)	31.4% (336)	0.003
Any TLR	39.3% (48)	30.3% (324)	0.01
*Other major secondary endpoints*			
Restricted survival time to first CD-TLR (days) through 60 months	1335.4 ± 56.7* (48)	1486.8 ± 16.3* (316)	0.01
Primary sustained clinical improvement at 36 months	48.1% (50/104)	61.5% (556/904)	0.01
Secondary sustained clinical improvement at 36 months	76.7% (69/90)	81.5% (693/850)	0.26
Sustained hemodynamic improvement at 36 months	44.0% (40/91)	49.1% (396/807)	0.38
Change in health status from baseline by EQ-5D index to 36 month	0.270 ± 0.443 (71)	0.119 ± 0.318 (752)	0.007
Walking impairment by WIQ to 36 months	82.6 ± 27.4 (72)	73.6 ± 30.8 (759)	0.02
Nights in hospital due to index lesion to 36 months	5.7 ± 11.8 (156)	2.7 ± 7.0 (1246)	0.002

For clinical safety endpoints, percentages are cumulative incidence based on the Kaplan–Meier Estimate (number of patients with events). Categorical data are given as the percentage (number/observations with data). Continuous data are presented as the mean ± standard deviation with the sample size unless otherwise stated. Adverse events were adjudicated by the independent Clinical Events Committee, all duplex ultrasound and angiographic measures were made by the independent core laboratories, and all other data were site reported. Definitions are described in Methods and Supplementary Methods

*Mean ± standard error

#### Participants with Both DM and CLTI

**Fig. 6:**
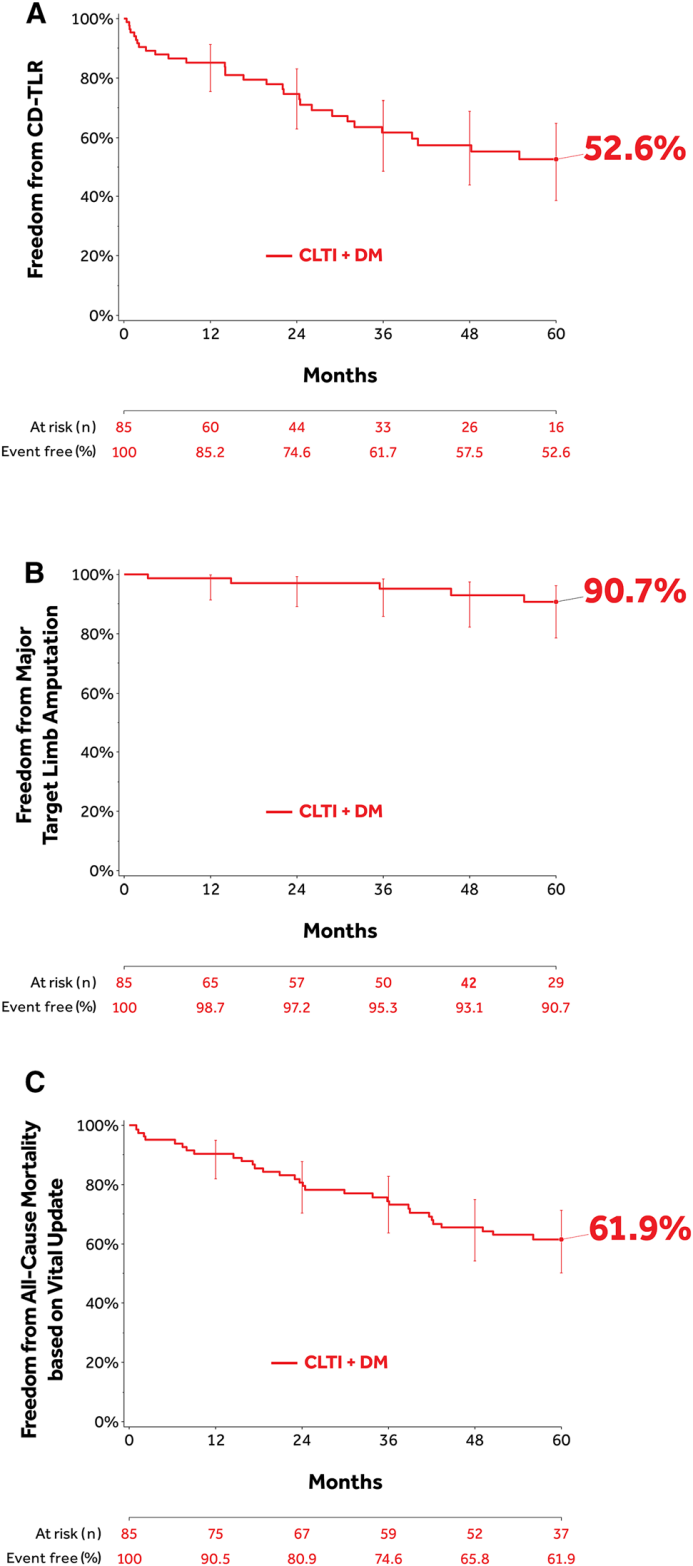
Subset analysis of participants with concomitant CLTI and DM in the IN.PACT Global Study. **(A)** Kaplan–Meier estimate of freedom from CD-TLR through 1800 days (60 months), **(B)** Kaplan–Meier estimate of freedom from major target limb amputation through 1800 days (60 months), and **(C)** Kaplan–Meier estimate of freedom from all-cause mortality after vital status update through 1800 Days (60 months). Bars represent the 95% confidence intervals. CLTI, chronic limb-threatening ischemia; DM, diabetes mellitus. CD-TLR, clinically driven target lesion revascularization.

Freedom from CD-TLR through 60 months was 52.6% (95% CI: 38.7–64.8%) in participants with concomitant CLTI and DM (Fig. 6A). The RMST to the first CD-TLR was 1254.9±80.0 days. Through 60 months, the freedom from major target limb amputation was 90.7% (95% CI: 78.5–96.1%) and freedom from all-cause mortality with vital status update was 61.9% (95% CI: 50.3–71.5%) (Fig. 6B and C).

## Discussion

This post hoc analysis evaluated the long-term clinical effectiveness of a DCB in patients with DM and/or CLTI compared to patients without those conditions. The strengths of this study included the prospective enrollment, rigorous adjudication of adverse events and high rates of compliance follow-up. Reintervention and amputation rates were low through 5 years, but expectedly higher in patients with CLTI compared to IC. Primary sustained clinical improvement through 36 months was achieved in over 50% of patients with DM or CLTI, although it was lower compared to non-DM and IC participants. Overall long-term survival was lower in patients with DM and CLTI, compared to non-DM and IC, highlighting the frailty of these patients [19–21].

DM is a risk factor for PAD and accelerated PAD progression leading to more ischemic events [22, 23]. Similarly, the present study observed a higher percentage of CLTI among DM compared to non-DM at baseline. DM patients also had more comorbidities, including obesity, hypertension, hyperlipidemia, and renal insufficiency, and more extensive vascular disease including more severe calcification and concomitant below-the-knee disease. Nonetheless, DCB angioplasty demonstrated good 5-year clinical outcomes in patients with DM, with similar freedom from CD-TLR as non-DM. There is a paucity of real-world femoropopliteal studies that reported 5-year effectiveness and safety outcomes of DCB in DM patients. A few registries (BIOLUX P-III and Lutonix Global SFA) analyzed DCB outcomes in DM subsets; however, outcomes were reported only through 2 years [15, 24]. Long-term interaction effects between DM status and treatment modality (DCB versus plain balloon angioplasty) were examined in the IN.PACT SFA and EffPac RCTs, showing no statistically significant interaction effects for CD-TLR (IN.PACT SFA) or primary patency (EffPac) between DM status and treatment modality [4, 25].

In the present analysis, the 5-year cumulative incidence of major amputation remained low in both DM (2.5%) and non-DM (1.1%). These findings are notable considering that a significant number of amputations occur every year due to diabetes-related complications [26]. The current results are also favorable compared to other endovascular studies of DM patients. In a prospective registry of 765 patients (560 DM, 205 non-DM) undergoing endovascular therapy for symptomatic PAD, the above-the-ankle amputation rates were 5.6% in DM and 3.3% in non-DM patients [19]. Conversely, a retrospective study reported 5-year limb salvage rates of 84% DM and 93% non-DM overall, and 72% DM and 79% non-DM in patients presenting with CLTI after PTA/stent infrainguinal revascularization [27].

Five-year all-cause mortality was significantly higher in patients with DM (23.8%) compared to non-DM (16.6%) in the present study. Mueller et al. reported 5-year mortality rates of 10% non-DM and 23% DM in PAD patients who are  < 75 years, and 38% non-DM and 52% DM in PAD patients who are ≥75 years [28]. These results were corroborated by a meta-analysis showing 5-year mortality rates ranging from 32 to 68% in DM patients versus 19 to 42% in non-DM patients (odds ratio 1.89, *p* < 0.001) with PAD [29]. In the present study, 44.5% of DM patients were insulin-dependent. The 5-year cumulative incidence of mortality with vital status update was significantly higher in the insulin-dependent sub-cohort compared to the non-insulin-dependent sub-cohort, and aligned with previous reports [30, 31]. A database analysis (*N* = 8022) reported a significantly increased risk of post-procedural mortality in insulin-dependent DM versus non-insulin-dependent DM patients (odds ratio 2.0, *p * =  0.009) [30].

In line with a prior report [32], CLTI participants had significantly higher baseline comorbidities than IC participants, as well as a higher incidence of long, calcified lesions. There was also more popliteal involvement in the CLTI compared to IC (41.8% versus 25.5%). This complexity was reflected in the significantly lower 5-year freedom from CD-TLR in CLTI (60.7%) compared to IC (70.5%). There are no published long-term TLR data after DCB angioplasty in CLTI patients. Therefore, the current comparisons are done with mixed populations consisting of both IC and CLTI. In a presentation, the 5-year freedom from CD-TLR was reported to be 68.5% and 70.3% in the DCB arms of the ILLUMENATE EU (mean lesion length 7.2 cm) and the ILLUMENATE Pivotal (mean lesion length 8.3 cm) RCTs [33]. However, those RCTs consisted of primarily IC patients with less complex lesions. Five-year freedom from CD-TLR was slightly higher in the AcoArt I RCT (77.5% in the DCB arm; mean lesion length 14.7 cm) [34] than the present study; however, AcoArt I DCB patients were younger, had less DM, and had fewer total occlusions (and calcification was not reported).

Despite the complexity, DCB angioplasty showed a sustained safety profile in the CLTI cohort. More than 50% of CLTI patients were free from the safety events through 5 years. In population-based studies, the long-term prognosis for CLTI patients is unfavorable, [35] with 5-year mortality rates higher than most cancers. A Medicare beneficiary study of 72,199 patients reported a 4-year mortality rate of 54% following CLTI diagnosis [36]. In a recent review article reporting on 4 to 5 years time horizons, mortality commonly exceeded 50%, but mortality was as high as 85% in patients >70 years undergoing amputation [35]. In the present study also, all-cause mortality was significantly higher in CLTI compared to IC (37.4% versus 17.4%). However, this rate is favorable compared to population-based studies, and is aligned with the 24.1–45.0% mortality rates reported for BEST-CLI and BASIL-2 trials at a median follow-up of 1.6-3.3 years after endovascular interventions [37, 38].

The 5-year major target limb amputation rates in the current study (6.8% CLTI, 1.1% IC) compare favorably to the 1.4%, 1.5%, and 2.3% rates in the DCB arms of the ILLUMENATE EU, ILLUMENATE Pivotal, and AcoArt I RCTs (33, 34), all of which enrolled primarily IC patients. At the time of writing this paper, no other global DCB studies have reported amputation rates through 5 years. In population-based studies, amputation rates are unacceptably high in CLTI patients, typically exceeding 15–20% at 1 year [35]. A prospective population-based study in the United Kingdom reported a 5-year amputation rate of 43.4% in CLTI patients, [20] while a pooled analysis from the Netherlands reported 5-year major amputation rates of 34.1% in CLTI patients with DM and 20.4% without DM [21]. Recently, the BEST-CLI trial reported above-ankle index-limb amputation rates of 14.2% to 14.9% at a median follow-up of 1.6 to 2.7 years after endovascular intervention [37]. However, a direct comparison between the present study and BEST-CLI is not possible due to differences in study design, endovascular modality (only 25–28% of BEST-CLI patients received a DCB), and patient demographics (more DM patients were included in BEST-CLI). Interestingly, in the present study, a subset analysis of CLTI patients with concurrent DM showed that 5-year freedom from major target limb amputation (90.7%) and freedom from all-cause mortality (61.9%) were not worse than the overall CLTI cohort, albeit with a lower rate of freedom from CD-TLR (52.6%), suggesting that while more reinterventions are required in this vulnerable subset, safety can be reasonably achieved.

An incremental increase in amputation rates with increasing RC has been well documented [35]. RC 6 was excluded in the present study, which may have contributed to the low major target limb amputation rate. Also, most patients were treated for RC 4. Nonetheless, the 6.8% 5-year major target limb amputation rate in CLTI patients (RC 4–5) with complex lesions is highly encouraging. Furthermore, there may be cost-benefit implications of DCB for CLTI patients. It has been shown that CLTI is associated with high healthcare costs [39]. A recent IN.PACT Global CLTI cost analysis reported that DCB treatment was associated with improved patient outcomes and significant cost savings in the Dutch and German healthcare systems [40]. The authors concluded that DCB is a cost-effective modality and likely the dominant treatment strategy for CLTI patients with femoropopliteal lesions.

### Limitations

This was a non-blinded study with no comparator arm. The CLTI cohort was relatively small, partially enrolled as the result of protocol deviations, and no hypotheses were pre-specified to assess statistical power. This CLTI cohort comprised only patients with RC 4 and RC 5; RC 6 was excluded from the enrollment. In the overall study, imaging data were not available for all patients hence no anatomic outcomes were analyzed in these cohorts.

## Conclusions

Results from this real-world study demonstrate encouraging 5-year reintervention and safety outcomes that are consistent with prior endovascular studies and the known increased risk profile of patients with DM and CLTI. DCB may be considered a treatment option for PAD patients with DM and/or CLTI; higher reintervention rates in patients with CLTI versus claudicants should be considered when determining follow-up plans.

### Supplementary Information

Below is the link to the electronic supplementary material.

Supplementary file1 (DOCX 187 kb)
