# Supplementary material for: Five-Year Outcomes after Paclitaxel Drug-Coated Balloon Treatment of Femoropopliteal Lesions in Diabetic and Chronic Limb-Threatening Ischemia Cohorts: IN.PACT Global Study Post Hoc Analysis
Source: Cardiovasc Intervent Radiol. 2023 Aug 1;46(10):1329–45. doi: 10.1007/s00270-023-03478-y (PMC10547845; doi:10.1007/s00270-023-03478-y)
Supplement: Supplementary file 1 — Supplementary file1 (DOCX 187 kb) [file 270_2023_3478_MOESM1_ESM.docx]

**SUPPLEMENTARY ONLINE MATERIALS**

**Supplementary Content List**

**Supplementary Methods**

**Supplementary Table 1.** List of Investigators Who Enrolled Participants in the IN.PACT Global Study

**Supplementary Figure 1.** Subset analysis of insulin-dependent and non-insulin dependent diabetic mellitus (DM) participants.

**Supplementary Methods**

Baseline data were reported by the investigational sites. Participant demographic and clinical characteristics were participant-based unless otherwise stated. Lesion and procedural characteristics were lesion-based except for the number of bilateral participants, days of hospital stay due to the index procedure, immediate hemodynamic improvement at post-index procedure, pre-dilation, and post-dilation (participant-based), or as otherwise stated in the definitions.

*Study endpoint definitions*

Severe calcification was defined as calcification with circumference ≥180° (both sides of the vessel at the same location) and a length greater than or equal to half of the total lesion length.

Diameter stenosis was defined as residual stenosis assessed based on visual estimate.(1)

Spot stenting was defined as use of the single shortest stent in which minimal length was sufficient to cover the residual stenosis but did not cover the entire original length of the target lesion.

Partial lesion coverage was defined as use of a stent length longer than the residual stenosis but shorter than the original length of the target lesion.

Immediate hemodynamic improvement is defined as an ABI improvement of ≥ 0.1 or to an ABI ≥ 0.9.

Device success was defined as successful delivery, inflation, deflation, and retrieval of the intact study balloon device without burst below the rated burst pressure. This analysis is device (balloon) based.

Procedural success was defined as residual stenosis ≤50% for non-stented patients or ≤30% for stented patients by visual estimate. This analysis is lesion based.

Clinical success was defined as procedural success without complications (death, major target limb amputation, thrombosis of the target lesion, or target vessel revascularization) prior to discharge. This analysis is participant based.

Primary sustained clinical improvement is defined as sustained upward shift of at least 1 category on Rutherford classification as compared to baseline without the repeated TLR or surgical revascularization in amputation-free surviving participants.

Secondary sustained clinical improvement is defined as sustained upward shift of at least 1 category on Rutherford classification as compared to baseline including the need for repeated TLR or surgical revascularization in amputation-free surviving participants.

Sustained hemodynamic improvement is defined as persistent improvement of ABI-values with ≥ 0.1 as compared to baseline values or to an ABI ≥ 0.9 throughout follow-up without the need for repeated TLR or surgical revascularization in amputation-free surviving participants.

Safety composite endpoint consists of freedom from device- and procedure-related death to 30 days, freedom from target limb amputation within 60 months; and freedom from CD-TVR within 60 months.

CD-TVR was defined as any reintervention within the target vessel due to symptoms or drop of ABI ≥20% or >0.15 when compared with the post procedure baseline ABI.

MAE composite is defined as all-cause death, clinically driven TVR, major target limb amputation, thrombosis at the target lesion site at 60 months.

CD-TLR is defined as any reintervention within the target lesion(s) because of symptoms or drop of ankle-brachial index (ABI) of ≥20% or >0.15 when compared with post-index procedure baseline ABI.

Health status assessed by EQ-5D questionnaire index at 36 months.

Walking impairment assessed by Walking Impairment Questionnaire (WIQ) at 36 months.

Nights in hospital due to index lesion at 36 months is cumulative days.

**References**

1. Dattilo R, Himmelstein SI, Cuff RF. The COMPLIANCE 360 degrees Trial: a randomized, prospective, multicenter, pilot study comparing acute and long-term results of orbital atherectomy to balloon angioplasty for calcified femoropopliteal disease. J Invasive Cardiol. 2014;26(8):355-60.

**Supplementary Table 1. List of Investigators Who Enrolled Participants in the IN.PACT Global Study**

| **Clinical Site** | **Location** | **Principal Investigator** |
| --- | --- | --- |
| Universitäts-Herzzentrum Freiburg - Bad Krozingen GmbH | Bad Krozingen, Germany | Thomas Zeller |
| St. Franziskus-Hospital Münster GmbH | Münster, Germany | Giovanni Torsello |
| RoMed Klinikum Rosenheim | Rosenheim, Germany | Gunnar Tepe |
| Imeldaziekenhuis | Bonheiden, Belgium | Patrick Peeters |
| Universitätsklinikum Leipzig AöR | Leipzig, Germany | Dierk Scheinert |
| AZ Sint-Blasius | Dendermonde, Belgium | Marc Bosiers |
| Onze-Lieve-Vrouwziekenhuis | Aalst, Belgium | Lieven Maene |
| Maria Eleonora Hospital | Palermo, Italy | Antonio Micari  Vincenzo Pernice |
| Inselspital Universitätsspital Bern | Bern, Switzerland | Dai-Do Do  Joern Fredrik Dopheide |
| Universitair Ziekenhuis Antwerpen | Edegem, Belgium | Jeroen Hendriks |
| RZ Tienen | Tienen, Belgium | Koen Keirse |
| Landeskrankenhaus - Universitätsklinikum Graz | Graz, Austria | Marianne Brodmann |
| Semmelweis Egyetem AOK | Budapest, Hungary | Bela Merkely |
| Rijnstate* | Arnhem, The Netherlands | Jan-Willem Lardenoije |
| Bács-Kiskun Megyei Kórháza | Kecskemét, Hungary | Zoltan Ruzsa |
| Universitätsklinikum Heidelberg | Heidelberg, Germany | Britta Vogel  Christian Erbel |
| AO Universitaria Policlinico Vittorio Emanuele Presidio G R | Catania, Italy | Pierfrancesco Veroux |
| Hosptial de Santa Marta | Lisbon, Portugal | Joao Albuquerque e Castro  Gonçalo Rodrigues |
| Hopital Cantonal HFR | Fribourg, Switzerland | Daniel Periard |
| Euromedic | Katowice, Poland | Tomasz Ludyga |
| Groupe hospitalier Pellegrin | Bordeaux Cedex, France | Dominique Midy |
| Severance Hospital | Seoul, South Korea | Donghoon Choi |
| Ziekenhuis Oost Limburg - Campus Sint-Jan | Genk, Belgium | Wouter Lansink |
| Universitätsklinikum Tübingen | Tübingen, Germany | Dominik Ketelsen  Gerd Grozinger |
| Royal Prince Alfred Hospital | Sydney, Australia | Steven Dubenec |
| Luzerner Kantosspital | Luzern, Switzerland | Martin Banyai  Thomas Bieri |
| Hôpitaux Universitaires de Strasbourg - Hôpital Civil | Strasbourg, France | Nabil Chakfe |
| Landesklinikum Thermenregion Mödling | Mödling, Austria | Franz Xaver Roithinger |
| Università Cattolica del Sacro Cuore Policlinico Gemelli | Rome, Italy | Carlo Trani |
| As-Salam International Hospital | Cairo, Egypt | Hossam Mansour |
| Korea University Guro Hospital | Seoul, South Korea | Seung-Woon Rha |
| Universitair Ziekenhuis Gent | Gent, Belgium | Frank Vermassen |
| Rabin Medical Center - Beilinson Hospital | Petach Tikva, Israel | Alexander Belenky |
| VUSCH | Kosice, Slovakia | Lubomir Spak  Matej Moscovic |
| Manchester Royal Infirmary | Manchester, United Kingdom | Nicholas Chalmers  Richard Hoddes |
| Centre Hospitalier Universitaire de Sherbrooke (CHUS) | Sherbrooke, Canada | Andrew Benko |
| Changi General Hospital | Singapore | Steven Kum |
| Ajou University Hospital | Seoul, South Korea | Je Hwan Won |
| SUSCCH | Banská Bystrica, Slovakia | Matej Vozar |
| Toronto General Hospital | Toronto, Canada | Kong Teng Tan |
| Egypt Air Hospital | Cairo, Egypt | Mamdouh Labib |
| Universitair Medisch Centrum Utrecht | Utrecht, The Netherlands | Gert-Jan de Borst |
| Samsung Medical Center | Seoul, South Korea | Young-Soo Do |
| Catharina Ziekenhuis | Eindhoven, The Netherlands | Joep Teijink |
| Clinica Santa Maria | Medellin, Antioguia, Columbia | Juan Fernando Gomez |
| Samodzielny Publiczny Szpital Kliniczny Nr 2 PAM w Szczecini | Szczecin, Poland | Aleksander Falkowski |
| Clinica La Sagrada Familia | Buenos Aires, Argentina | Luis  Mariano Ferreira |
| University Medical Centre Maribor | Maribor, Slovenia | Jozef Matela |
| Asan Medical Center | Seoul, South Korea | Seung-Whan Lee |
| Jeroen Bosch Ziekenhuis | Hertogenbosch, The Netherlands | Bart Verhoeven |
| Carmel Medical Centre | Haifa 34362, Israel | Dalit Mannheim |
| Azienda Ospedaliera Ordine Mauriziano | Torino, Italy | Franco Nessi |
| Narodny ustav srdcovych a cievnych chorob, a.s. (NUSCH) | Bratislava, Slovakia | Ivan Vulev  Stefan Pataky |
| St. Antonius Ziekenhuis | Nieuwegein, The Netherlands | Jean-Paul de Vries |
| Faculty Hosptial Hradec Kralove | Hradec Kralove, Czech Republic | Radovan Maly |
| City Clinical Hospital #71 | Moscow, Russia | Zaza Kavteladze |
| Sheffield Vascular Institute, Sheffield Teaching Hospitals | Sheffield, United Kingdom | Douglas Turner |
| Fundacion Favaloro | Buenos Aires, Argentina | Oscar Mendiz |
| Augusta Krankenhaus | Duesseldorf, Germany | Ralf Kolvenbach |
| General University Hospital of Patras | Patra, Greece | Dimitrios Karnabatidis |
| Clinica Medilaser | Huila, Columbia | Cesar Cuellar |
| Helsingin Seudun Yliopistollinen Keskussairaala | Helsinki, Finland | Maarit Venermo |
| Kaunas Medical University Clinic | Kaunas, Lithuania | Linas Velicka |
| Karolinska Universitetssjukhuset | Solna, Sweden | Goran Lundberg |

* Michel Reijnen, MD, PhD is a co-investigator at this site


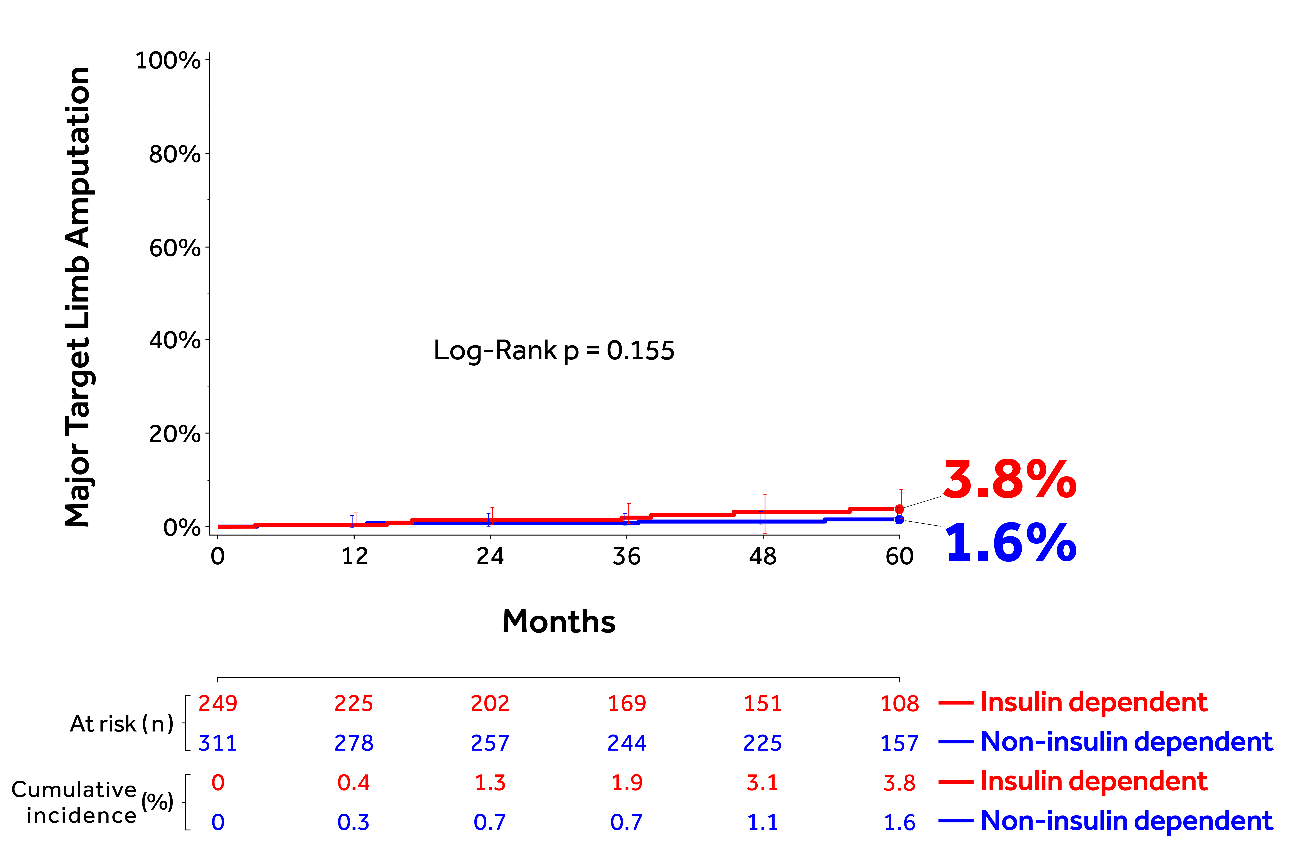

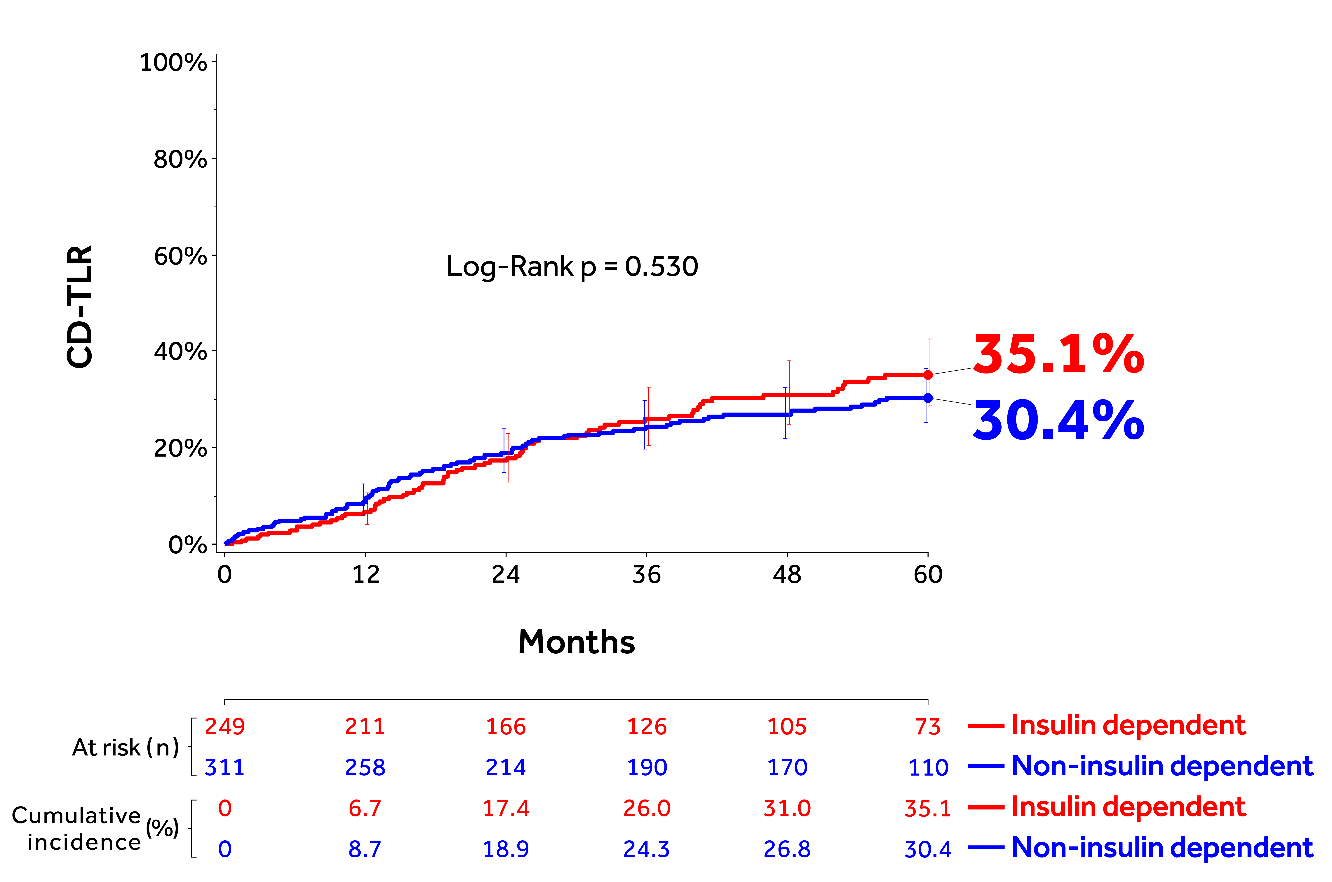


**B**

**A**

**A**

**C**


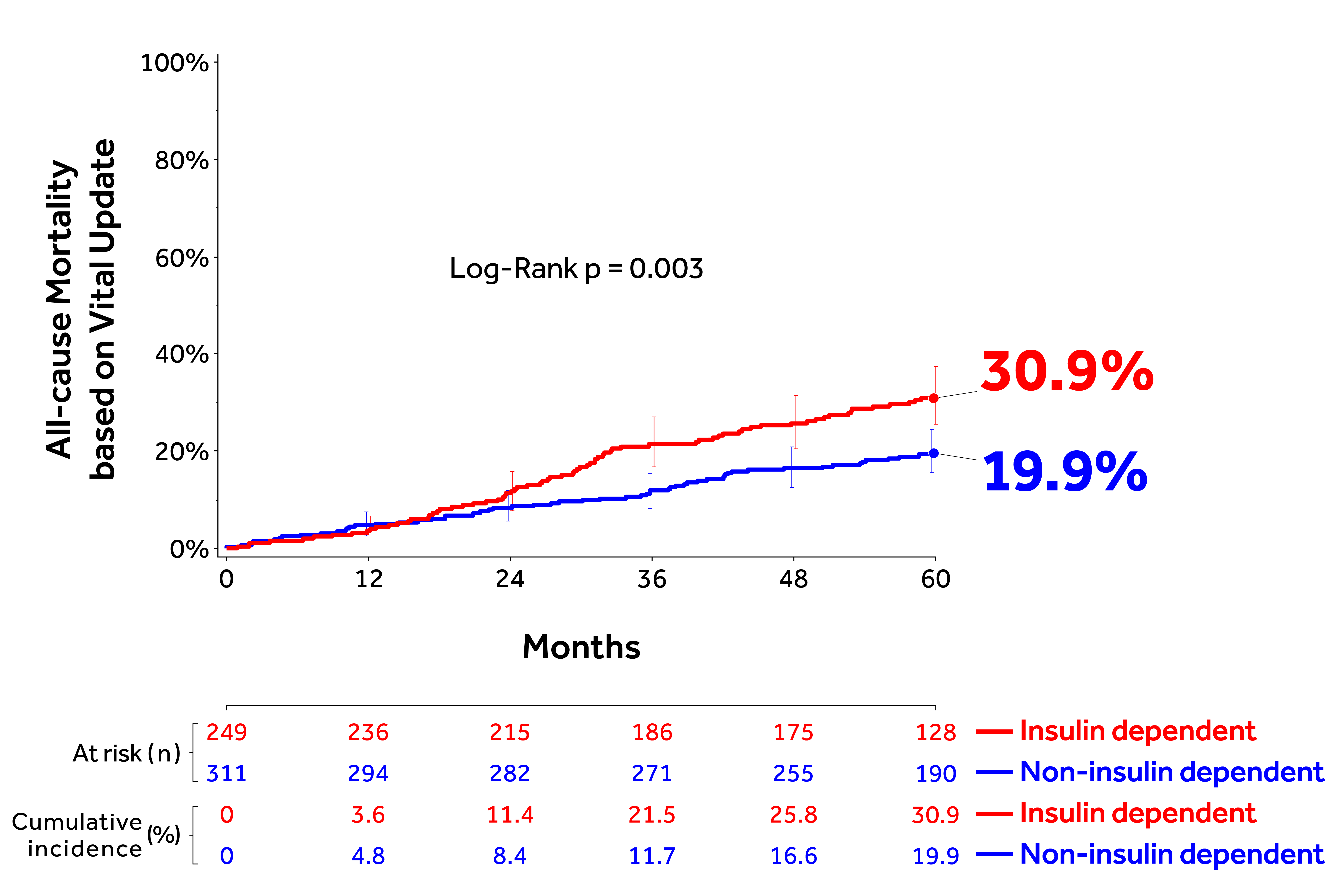


**Supplementary Figure 1.** Subset analysis of insulin-dependent and non-insulin dependent diabetic mellitus (DM) participants. Kaplan-Meier cumulative incidence of **A)** clinically driven target lesion revascularization (CD-TLR), **B)** major target limb amputation, and **C)** all-cause mortality after vital status update through 1800 days (60 months) in the insulin dependent and non-insulin dependent DM subcohorts in the IN.PACT Global Study. Bars represent the 95% confidence intervals.
